# Diabetes Prevention in Hispanics: Report From a Randomized Controlled Trial

**DOI:** 10.5888/pcd11.130119

**Published:** 2014-02-27

**Authors:** Catherine Duggan, Elizabeth Carosso, Norma Mariscal, Ilda Islas, Genoveva Ibarra, Sarah Holte, Wade Copeland, Sandra Linde, Beti Thompson

**Affiliations:** Author Affiliations: Elizabeth Carosso, Norma Mariscal, Ilda Islas, Genoveva Ibarra, Sarah Holte, Wade Copeland, Fred Hutchinson Cancer Research Center, N Seattle, Washington; Sandra Linde, Sunnyside Community Hospital, Sunnyside, Washington; Beti Thompson, Fred Hutchinson Cancer Research Center and University of Washington, Seattle, Washington.

## Abstract

**Introduction:**

Hispanics are at increased risk of developing type 2 diabetes. Lifestyle interventions are effective in preventing diabetes and restoring glucose regulation.

**Methods:**

We recruited Hispanic men and women (N = 320) who were residents of the Lower Yakima Valley, Washington, aged 18 years or older with hemoglobin A1c (HbA1c) levels higher than 6% to a parallel 2-arm randomized-controlled trial conducted from 2008 through 2012. The trial compared participants in the intervention arm, who received an immediate educational curriculum (n = 166), to participants in the control arm, who received a delayed educational curriculum (n = 154). The home-based curriculum consisted of 5 sessions led by community health workers and was designed to inform participants about diabetes, diabetes treatment, and healthy dietary and physical activity behaviors. Participants were randomly assigned to the intervention and control arms, and analysts were blinded as to participant arm. We evaluated intervention effects on HbA1c levels; frequency (times per week) of fruit and vegetable consumption; and frequency (times per week) of mild, moderate, and strenuous leisure-time physical activity. At baseline, 3 months, and 6 months after randomization, participants completed a questionnaire and provided a blood sample. Analysts were blinded to intervention arm.

**Results:**

The immediate intervention group (−0.64% [standard error (SE) 0.10]) showed a significant improvement in HbA1c scores (–37.5%, *P* = .04) compared with the delayed intervention group (–0.44%, *P* = .14). No significant changes were seen for dietary end points or changes in physical activity. We did observe a trend of greater increases in frequency of moderate and vigorous physical activity and a smaller increase in mild physical activity in the immediate intervention group than in the delayed intervention group.

**Conclusion:**

This home-based intervention delivered by CHWs was associated with a clinically and statistically significant reduction in HbA1c levels in Hispanic adults with HbA1c levels higher than 6%.

## Introduction

Type 2 diabetes is a chronic metabolic disease characterized by insulin resistance and elevated blood glucose levels. Diagnosis is based on glycated hemoglobin (HbA1c) levels higher than 6.5%, or fasting plasma glucose levels higher than 126 mg/dL (1). Prevalence and incidence varies by ethnicity: Hispanics have a greater risk of developing type 2 diabetes (2–5). The population age-adjusted incidence of diabetes in 2007–2009 for people aged 20 years or older was 7.1% for non-Hispanic whites and 11.8% for Hispanics, corresponding to a 66% higher risk (6). Prediabetes, characterized by higher-than-normal blood glucose and increased risk of developing type 2 diabetes, also disproportionately affects Hispanics (7). Hispanics are more likely to have poor glycemic control (8), are less likely to use diabetes medical and self-management practices, such as obtaining regular medical check-ups and self-monitoring of glucose levels, and are more likely to experience complications (9–11).

Lifestyle interventions can prevent type 2 diabetes in at-risk populations by improving glycemic control. The Diabetes Prevention Program (DPP), a large randomized controlled trial (RCT) of people at high risk for diabetes, demonstrated that a behavioral lifestyle intervention to lose weight and increase physical activity (PA) reduced development of type 2 diabetes by 58% during a 3-year period (12). Although lifestyle interventions are more cost-effective than medications, reports identified difficulties in disseminating the original DPP intervention, including relatively high cost of one-on-one delivery by behavioral experts, challenges in implementing it in busy health care settings, and Medicare’s lack of coverage for diet and exercise programs to prevent diabetes in people with prediabetes (13). The DPP oversampled minorities, but only 16% of its participants were Hispanics, who were mostly well-educated and of higher socioeconomic status. However, interventions successful in one racial/ethnic group may not be generalizable to others because of specific cultural or economic barriers. For example, Hispanics are more likely than other groups to lack regular health care sources, to use hospital emergency departments or public health clinics (14), and to experience cultural and economic barriers to care, including the inability to speak English and low rates of health insurance coverage. Thus, a 6-month intervention aimed at Hispanics and held exclusively in physicians’ offices may recruit too few members of vulnerable, underserved low-income groups (15).

The purpose of this study was to evaluate the effect of an RCT of a home-based educational intervention administered by community health workers (CHW, or *Promotores de Salud* in Spanish) for Hispanic people with elevated HbA1c levels (>6%) in the Yakima Valley, a rural area where more than 60% of the population is Hispanic and low-income. Specific aims of the project were to compare the effects of the immediate intervention with those of the delayed intervention on changes in 1) HbA1c levels before and after the intervention; 2) fruit and vegetable consumption, assessed by self-reported questionnaire; and 3) frequency (times per week) of mild, moderate, or vigorous leisure-time PA assessed by self-reported questionnaire.

## Methods

Recruitment of participants has been described previously (16). Hispanic men and women aged 18 or older who lived in the Lower Yakima Valley, Washington, were recruited at health fairs and local community events held from 2008 through 2012. Sunnyside Community Hospital provided free blood glucose screening at these events. CHWs approached attendees to introduce the study and assess attendees’ interest in participating before giving the blood test. Although participation was not restricted to Hispanics of Mexican origin, 95.9% of Hispanics who live in the Yakima Valley report being of Mexican origin (17).

Interested people completed a screening survey and provided written informed consent to have blood glucose levels only measured via a fingerstick blood sample. People who received the free blood glucose test were not required to participate in the study. Fasting/nonfasting criteria based on participants’ self-report of food intake in the last 8 hours were used to interpret results. Those with abnormal glucose results (>110 mg/dL if fasting) were referred to local hospitals for free fasting blood glucose and HbA1c testing, which acted as the baseline HbA1c for study participants.

Interested participants provided a second written informed consent for study participation and completed a baseline questionnaire. Participants were randomly assigned to an immediate or a delayed intervention. CHWs were aware of participants’ arm; outcome analysts were blinded. 

**Figure 1:**
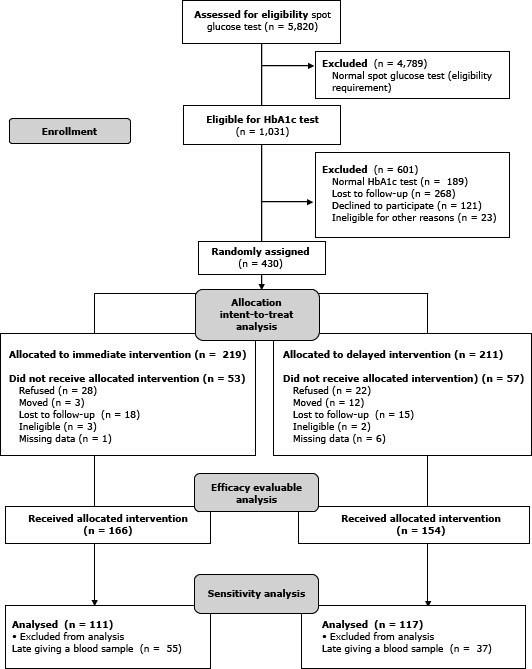
Overview of the process for the randomized-controlled prevention trial of the Partnership for a Hispanic Diabetes Prevention Program.

The number of Hispanics screened was 5,820; 17.7% (n = 1,031) had abnormal spot glucose results. Of these, 601 had normal HbA1c levels, were ineligible, refused participation, or were lost to follow-up (Figure 1). The remaining 430 participants (intention-to-treat group) were randomly assigned to either the immediate intervention (n = 219) or the delayed intervention (n = 211). Of these, 50 participants (28 immediate; 22 delayed) refused the intervention after randomization; 15 (3 immediate, 12 delayed) moved and could not participate; 33 (18 immediate, 15 delayed) were lost to follow-up; 5 (3 immediate, 2 delayed) were found to be ineligible after randomization; and 7 (1 immediate, 6 delayed) had significant missing data. Remaining participants (n = 320) represent the sample for whom the effectiveness of the intervention could be assessed (efficacy evaluable [EE] sample). A further 92 participants (28.8%) had a follow-up blood draw, but not within the scheduled time frame, and were thus excluded as part of a sensitivity analysis, leaving a total of 228 participants (n = 111 immediate; n = 117 delayed).

The intervention consisted of 5 guided educational sessions conducted in participants’ homes. Trained bilingual Hispanic CHWs employed at the Center for Community Health in Sunnyside, Washington, presented the sessions on diabetes over the course of 5 weeks at scheduled times (1 per week). Sessions were rescheduled occasionally to meet the participants’ requirements. Each session lasted approximately 1 hour. At each session, the CHW presented an educational curriculum involving diabetes education and awareness and methods to increase self-management of diabetes (18). Participants were encouraged to invite family members and friends to participate, to educate them about the importance of healthy lifestyle in diabetes management so they could be supportive of the participants. Discussion was facilitated by a set of flip charts developed by investigators with input from the community, revised, and translated into Spanish. CHWs were trained in both diabetes education and in working with the community (100 hours of training, including CDC’s Community Health Worker Evaluation Tool Kit, and training by a local diabetes specialist). They received twice-yearly refresher courses during the study. The intervention was offered in English or Spanish and was presented in easy-to-understand language. It was developed by using social cognitive theory constructs based on selected cultural symbols and themes, cultural patterns and concepts, values, norms, and relationships to promote healthy lifestyles. The intervention was developed via soliciting feedback from Hispanic participants in the Valley. The community advisory board, made up of members of the community, worked with investigators to select an appropriate intervention. They gave final approval for implementing the intervention in the community. In addition, we held numerous focus groups with Hispanic residents of this area to test the intervention for cultural relativity before it was finalized.

Session topics were 1) general diabetes (function of insulin, types of diabetes, symptoms and control of diabetes, risk, screening and diagnosis); 2) self-management (treatment, blood glucose monitoring, HbA1c test); 3) diet (carbohydrates and counting, healthy eating, serving sizes, food labels, nutrition facts); 4) physical activity (recommended levels, benefits, pedometers and steps per day); and 5) complications of diabetes. In sessions 2 through 4, presenters reviewed the main points from previous sessions before introducing a new topic. Each session ended with a summary of the presentation. Group discussion was encouraged. Participants were given the opportunity to ask questions throughout the presentation. Participants were also given placemats that show portion sizes, measuring cups, a diabetes cookbook with recipes for Mexican cuisine, and various pictures and reminders about diabetes. Study procedures were reviewed and approved by the Fred Hutchinson Cancer Research Center Institutional Review Board (file number 6194). This study is registered at http://clinicaltrials.gov/show/NCT01564797.

Primary end points were measured at baseline and 3 months postrandomization. Participants in the delayed intervention group were then given the intervention. A third blood sample was collected from all participants and a third questionnaire administered at the 6-month time point.

Primary outcomes of our study were 1) changes in serum levels of HbA1c from baseline to 3 months; 2) changes in fruit and vegetable consumption from baseline to 3 months assessed by self-report using the 5 A Day for Better Health Program, validated questionnaires (19,20) that asked about usual frequency of consumption (times per week) of fruit juice, sugar-sweetened beverages, and fresh fruit and vegetables and cooking habits (eg, frequency of eating out vs cooking at home and types of fats used in cooking); and 3) changes in frequency (times per week) of mild, moderate, or vigorous leisure-time PA from baseline to 3 months assessed by self-reported questionnaire adapted from the International Physical Activity Questionnaire (IPAQ) (21), completed at baseline, and at 3-month and 6-month time points. The questionnaire also asked whether participants engaged in PA as part of paid employment.

**Figure 2:**
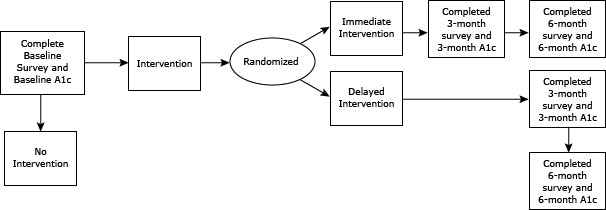
Timing of intervention and HbA1c measurements. Abbreviation: HbA1c, hemoglobin A1c.

Secondary outcomes compared changes in HbA1c levels in the delayed intervention arm, comparing changes at 3 months with baseline levels and levels at 6 months with 3-month levels. Participants in the delayed intervention (control) arm received the educational program 6 months following initiation of the study (Figure 2). Finally, we investigated whether participants in the intervention arm maintained improved blood glucose control at 6 months.

Our study size of 320 participants (EE group) had 80% power to detect a 29% difference in mean HbA1c levels by using a 1-sided *t* test with significance of .05%. Primary outcomes analyzed changes in the variables of interest from baseline to 3 months in the immediate intervention arm, compared with change in the delayed intervention arm. Continuous normally distributed data were analyzed by using the Student *t* test. For non-normally distributed data, the Wilcoxon Rank-Sum test was applied, allowing for the possibility of ties. Categorical data were analyzed by using Pearson’s χ^2^ or Fisher’s exact test. All tests were 1-sided and were performed using SAS version 9.1 (SAS Institute Inc, Cary, North Carolina).

## Results

Mean age at baseline was 50.6 years (Table 1). Women made up 70.6% of the EE group; 65.6% of participants had an 8th-grade education or less, and 61.8% of participants were unemployed at randomization. Mean body mass index of participants was 32.9 kg/m^2^, and 67.3% had been diagnosed with diabetes before randomization. There were no significant differences in any baseline participant characteristics between the intent-to-treat group (n = 430) and the EE group (N = 320; data not shown). There were no significant differences between baseline characteristics in the immediate vs the delayed intervention. Mean HbA1c levels for the immediate vs delayed intervention groups were 8.04% (standard error [SE], 0.17, and 8.31% (SE, 0.13), respectively (*P* = .12). At baseline, participants in the delayed intervention arm reported that they were taking either insulin (15.6%) or other medications (76.0%) for the treatment of diabetes; in the immediate intervention arm, 20.9% were taking insulin and 27.8% were taking other medications. Between baseline and 3 months, 1 participant in the delayed arm began taking insulin after randomization, compared with 5 participants in the immediate intervention arm; 2 participants in the immediate intervention group and 5 in the delayed intervention group stopped taking insulin. These differences were not significant (*P* = .25).

**Table 1:** Baseline Characteristics of the Intent-to-Treat Sample (N = 320)

Characteristic	All^a^	Immediate Intervention	Delayed Intervention	*P* b

	N = 320	n = 154	n = 166	
Age in years, mean (SE)	50.6 (1.0)	50.3 (1.0)	50.8 (1.0)	.75
Baseline HbA1c, % (mean SE)	8.18 (0.15)	8.04 (0.17)	8.31 (0.13)	.12
Body mass index, % (mean SE)	32.90 (0.89)	33.37 (1.01)	32.42 (0.77)	.29
**Sex, n (%)**				
Male	93 (29.43)	47 (30.7)	46 (28.2)	.72
Female	223 (70.56)	106 (69.3)	117 (71.8)	
**Education, n (%)**	**n = 314^c^**	**n = 152^c^**	**n = 162^c^**	.19
4th grade or less	111 (35.4)	48 (31.6)	63 (38.9)	
5th through 8th grade	95 (30.3)	46 (30.6)	49 (30.3)	
9th through 12th grade, no diploma	50 (15.9)	25 (16.5)	25 (15.4)	
High school graduate or GED	37 (11.8)	24 (15.8)	13 (8.0)	
Some college through associate degree	13 (4.1)	4 (2.6)	9 (5.6)	
Bachelor’s degree or more	8 (2.6%)	5 (3.3%)	3 (1.9%)	
**Employment status, n (%)**	**n = 314^c^**	**n = 151^c^**	**n = 163^c^**	.40
Full time	96 (30.6)	42 (27.8)	54 (33.13)	
Part time	24 (7.6)	14 (9.3)	10 (6.3)	
Not employed	194 (61.8)	95 (62.9)	99 (60.74)	
**Prior diagnosis of diabetes, n (%)**	**n = 315^c^**	**n = 152^c^**	**n = 163^c^**	.16
Yes	212 (67.3)	96 (63.2)	116 (71.2)	
No	103 (32.7)	56 (36.8)	47 (28.8)	

Abbreviations: SE, standard error; HbA1C, hemoglobin A1C; GED, general educational development.

a Differences in totals are due to missing data.

b *P* values for differences between groups; Pearson’s χ^2^.

c Alternative hypothesis states that the change from baseline to 3-month follow-up in the delayed intervention is greater than the change from baseline to 3-month follow-up in the immediate intervention. Categorical data were analyzed by using Pearson’s χ^2^ or Fisher’s exact test. All tests were 1-sided.

We demonstrated a significant reduction in HbA1c levels in the immediate versus delayed intervention for the EE baseline (n = 320); mean HbA1c scores were 8.04% (SE, 0.17) for the delayed intervention group and 8.31% (SE, 0.13) for the immediate group. At 3 months, the scores were 7.59% (SE, 0.13) for the delayed intervention group and 7.68% (SE, 0.11) for the immediate intervention group, resulting in an estimated change from baseline to 3 months of 0.44% (SE = 0.14) in the delayed intervention control and 0.64% (0.10) in the immediate intervention group (*P* = .04 for change in HbA1c levels by arm). We observed a significant result for the sensitivity analysis group (n = 128) (*P* = .05; data not shown), because of a reduction in power caused by the smaller numbers analyzed (ie, analyzing only those participants whose data were collected within the specified time frame); mean changes in both groups were similar.

We evaluated changes in HbA1c levels for delayed intervention controls who received the intervention after the initial 3-month period had elapsed. These participants’ HbA1c levels increased between 3 and 6 months (postintervention), compared with changes observed between baseline and 3 months; HbA1c scores declined by .44% from baseline to 3 months (8.04% [SE,0.17] to 7.59% [SE, 0.14], respectively) and increased by .09% from 3 months to 6 months (7.59% [SE, 0.13] to 7.69% [SE, 0.14], respectively) postintervention. For the immediate intervention group, there was not sufficient evidence to reject the null hypothesis that the 3-month measure was greater than the 6-month measure, indicating that participants who had the intervention in the first 3 months (n = 166) maintained their HbA1c reduction at 6 months (7.68% [0.11] vs 7.66% [0.15]; *P* = .64). We observed no significant change in fruit and vegetable consumption or in soft drink consumption between the 2 arms.

There was no significant change in frequency of leisure-time PA between the 2 groups (Table 2a). We did observe a trend of a greater increase in frequency of moderate and vigorous PA, and a smaller increase in mild PA in the immediate intervention group (change −0.56; −0.74; −0.11 for vigorous, moderate, and mild PA, respectively, where a negative value implies an increase) compared with the delayed intervention group (change −0.13, −0.41, and −0.45 respectively), comparing 3-month follow-up survey responses to baseline responses (Table 2b). People in the immediate intervention group who engaged in vigorous PA as part of paid employment were more likely to increase their leisure-time vigorous PA than people in the delayed intervention group (*P* = <.001, for change −1.26 [immediate] vs 1.05 [delayed], where a negative value implies an increase in frequency of leisure-time PA) (Table 2c).

**Table 2a:** Change in Weekly Frequency of Mild, Moderate, and Vigorous Leisure-Time Physical Activity for All Participants, From Baseline to 3 Months, by Intervention Arm

Leisure-Time Physical Activity	Delayed Intervention (n = 154)			Immediate Intervention (n = 166)			*P* c

	Baseline (n = 143)^a^	3 Months (n = 143)^a^	Δ^b^	Baseline (n = 159)	3 Months (n = 159)	Δ^b^	

	Mean (SE)						
Vigorous	0.59 (0.1)	0.72 (0.1)	−0.13 (0.2)	0.35 (0.1)	0.96 (0.2)	−0.56 (0.2)	.09
Moderate	1.27 (0.2)	1.76 (0.2)	−0.41 (0.2)	1.39 (0.2)	2.13 (0.2)	−0.74 (0.2)	.13
Mild	3.16 (0.2)	3.67 (0.2)	−0.45 (0.3)	3.43 (0.2)	3.59 (0.2)	−0.11 (0.2)	.72
Total	5.03 (0.4)	6.14 (0.4)	−0.99 (0.5)	5.16 (0.3)	6.69 (0.4)	−1.4 (0.4)	.29

Abbreviations: SE, standard error.

a Differences in totals are due to missing data.

b Δ: mean (SE) is the difference in times per week of leisure-time physical activity from baseline to 3-month measures. A negative value indicates an increase in frequency of exercise.

c Alternative hypothesis states that the change from baseline to 3-month follow-up in the delayed intervention is greater than the change from baseline to 3-month follow-up in the immediate intervention. Categorical data were analyzed by using Pearson’s χ^2^ or Fisher’s exact test. All tests were 1-sided.

**Table 2b:** Change in Weekly Frequency of Mild, Moderate, and Vigorous Leisure-Time Physical Activity for Participants Who Performed Vigorous Physical Activity at Work, Baseline to 3 Months, by Intervention Arm

Leisure-Time Physical Activity, Times per Week	Delayed Intervention (n = 21)			Immediate Intervention (N = 31)			***P* c**

	Baseline (n = 20)^a^	3 Months (n = 20)^a^	Δ^b^	Baseline (n = 31)	3 Months (n = 31)	Δ^b^	

	Mean (SE)						
Vigorous	2.10 (0.6)	0.9 (0.4)	1.05 (0.7)	0.58 (0.3)	1.84 (0.4)	−1.26 (0.3)	<.001
Moderate	2.52 (0.6)	3.5 (0.5)	−0.85 (0.7)	1.45 (0.4)	2.1 (0.5)	−0.65 (0.4)	.61
Mild	3.14 (0.7)	3.55 (0.6)	−0.25 (0.9)	3.00 (0.5)	2.48 (0.5)	0.52 (0.6)	.78
Total	7.76 (1.5)	7.95 (1.2)	−0.05 (1.7)	5.03 (0.9)	6.42 (1.1)	−1.39 (0.9)	.35

Abbreviation: SE, standard error.

a Differences in totals are due to missing data.

b Δ: mean (SE) is the difference in times per week of leisure-time physical activity from baseline to 3-month measures. A negative value indicates an increase in frequency of exercise.

c Alternative hypothesis states that the change from baseline to 3-month follow-up in the delayed intervention is greater than the change from baseline to 3-month follow-up in the immediate intervention. Categorical data were analyzed by using Pearson’s χ^2^ or Fisher’s exact test. All tests were 1-sided.

**Table 2c:** Change in Weekly Frequency of Mild, Moderate, and Vigorous Leisure-Time Physical Activity for Participants Who Did Not Perform Vigorous Physical Activity at Work, Baseline to 3 Months, by Intervention Arm

Leisure-Time Physical Activity, Times per Wk	Delayed Intervention (n = 131)			Immediate Intervention (n = 130)			*P* c

	Baseline (n = 122)^a^	3 months (n = 122)^a^	Δ^b^	Baseline (n = 128)^a^	3 months (n = 128)^a^	Δ^b^	

	Mean (SE)						
Vigorous	0.35 (0.1)	0.7 (0.2)	−0.32 (0.2)	0.29 (0.1)	0.68 (0.2)	−0.39 (0.2)	.73
Moderate	1.03 (0.2)	1.48 (0.2)	−0.39 (0.2)	1.38 (0.2)	2.08 (0.2)	−0.76 (0.2)	.11
Mild	3.11 (0.3)	3.65 (0.3)	−0.51 (0.3)	3.5 (0.2)	3.8 (0.2)	−0.26 (0.3)	.59
Total	4.5 (0.4)	5.83 (0.4)	−1.21 (0.5)	5.17 (0.4)	6.56 (0.4)	−1.41 (0.4)	.35

Abbreviations: SE, standard error.

a Differences in totals due to missing data.

b Δ: mean (SE) is the difference in times per week of leisure-time physical activity from baseline to 3-month measures. A negative value indicates an increase in frequency of exercise.

c Alternative hypothesis states that the change from baseline to 3-month follow-up in the delayed intervention is greater than the change from baseline to 3-month follow-up in the immediate intervention. Categorical data were analyzed by using Pearson’s χ^2^ or Fisher’s exact test. All tests were 1-sided.

## Discussion

Five sessions of a home-based educational curriculum led by CHWs resulted in significant and clinically meaningful reductions in mean levels of HbA1c. Participants who received the immediate intervention successfully maintained reductions in HbA1c levels at 6 months.

Evidence suggests improvements in diet and increased PA levels can prevent or delay development of type 2 diabetes and are effective regardless of race/ethnicity. A systematic review with meta-analysis examined whether RCTs based on culturally appropriate health education were more effective than usual health education for people with type 2 diabetes from ethnic minority groups. Although few studies fitted the selection criteria and were heterogeneous in methodologies and outcome measures, culturally appropriate health education in general was more effective than usual health education in reducing HbA1c levels and increasing knowledge (22).

CHWs and educational curricula promoting behavior change have been shown to be effective in educating communities about diabetes and in improving glycemic control. One 12-month community-based RCT of a lifestyle intervention (3 in-home meetings, and 13 group-sessions) in 312 Hispanics at risk for developing type 2 diabetes resulted in significant reductions in HbA1c levels (*P* = .009), compared with usual care (23). An intensive RCT of 3 months of weekly instruction followed by 6 months of biweekly support group sessions promoting behavior change also found significant reductions in mean HbA1c versus reductions in controls (24). The REACH Detroit Partnership enrolled 54 Hispanic adults with diabetes in five 2-hour group meetings delivered every 4 weeks, and reported significant improvements in HbA1c levels in the intervention group (*P* < .001) compared with usual care (*P* = .160) (25). At the Texas–Mexico border, a CHW-led program of culturally competent diabetes self-management education led to a nonsignificant reduction in HbA1c levels in 256 Mexican-Americans (24), and another (N = 150, 80% Hispanic females) led to significant reductions in HbA1c levels and increased diabetes knowledge at 6 months; this intervention was culturally specific and consisted of participative group education, telephone contact, and follow-up (26). Finally, 189 Hispanic patients, newly diagnosed with type 2 diabetes, were randomly assigned to a CHW-led intervention, case management, or standard provider care. After 6 months, participants in the CHW group had reduced use of emergency departments and greater improvements in health status, dietary habits, physical activity, and medication adherence (27).

We found no significant changes in frequency of eating out or in consumption of fresh fruits and vegetables or nonalcoholic beverages. This may be due to the self-reported nature of our measures. Furthermore, we did not capture serving sizes in the questionnaire, and our observed significant changes in HbA1c may be attributable to reduced serving sizes. Similarly, changes in frequency of leisure-time PA were not significant between the 2 groups but may have been underreported or misreported. In addition, we did not record the length of time that participants were involved in PA. However, we did observe a trend of a greater increase in moderate and vigorous PA and a smaller increase in mild PA in the immediate intervention group compared with the delayed intervention group from baseline to 3 months. Also, people who recorded performing vigorous PA at work were significantly more likely to increase the number of times per week they engaged in strenuous PA after the intervention than those who had more sedentary occupations.

Our intervention is appropriate for low-income minority groups. It is relatively short (5 weeks) and home-based, increasing the likelihood of participant adherence by removing barriers such as transportation. Bilingual CHWs removed linguistic barriers. Once the intervention was complete, we randomly selected 40 participants and conducted open-ended, structured interviews, and used qualitative methods to analyze transcripts. Participants showed high program satisfaction and interest, and almost all reported making at least 1 positive behavior change as a result of the intervention.

Previous research on education by CHWs in the Hispanic community has generally taken place in group or community settings, and few have been conducted in homes. Home-based education sessions are thought to be effective in reaching underserved individuals who may be unable to receive clinic-based services because of barriers such as lack of transportation, childcare, or language (28). One report of a home-based CHW intervention to promote healthy behaviors among Hispanic families (29) demonstrated significant increases in self-efficacy for changing food (*P* < .001) and PA (*P* < .001) behaviors.

Limitations of our study include the homogeneous population, which may limit generalizability of the study, and the use of self-reported questionnaires to measure change in diet and PA, which can be affected by recall bias and education level. The questionnaires did not capture food serving sizes, or the length of time PA was performed, so it is difficult to evaluate whether changes in these parameters are responsible for changes in HbA1c levels. However, as discussed earlier, we did see a change in the distribution of participants who performed moderate activity or vigorous activity from mild activity in the immediate compared with the delayed intervention, though this finding was not significant. We did not analyze the likelihood that participants who performed mild leisure-time activity transitioned to moderate or vigorous PA, for example, because an analysis of this complexity was outside the scope of this study. Strengths of the study include the randomized nature of the study; the relatively large sample size, and the adherence to the study: of the 430 participants randomized, 320 (74.4%) completed the study and gave a blood sample at all time points. The study also successfully focused on an underserved population. The results of this trial show that this intervention was successful in significantly reducing HbA1c levels in Hispanic Americans who live in the Yakima Valley.
